# NON-UNIONS AFTER FIXATION OF HUMERAL FRACTURES USING HACKETHAL'S BUNDLE NAILING TECHNIQUE

**DOI:** 10.1590/1413-785220162405150468

**Published:** 2016

**Authors:** PETR OBRUBA, STEFAN RAMMELT, LUBOMIR KOPP, KAREL EDELMANN, JAKUB AVENARIUS

**Affiliations:** 1. Clinic of Traumatology, Masaryk Hospital, Usti nad Labem, Czech Republic.; 2 >. 3rd Medical Faculty, Charles University, Prague, Czech Republic.; 3. University Center for Orthopaedics and Traumatology, Carl-Gustav Carus University Hospital, Dresden, Germany.

**Keywords:** Humeral fractures. Fracture fixation, intramedullary. Fracture fixation, internal.

## Abstract

**Objective:**

The aim of the study was to identify factors contributing to the development of non-union after fixation of diaphyseal humeral fractures using Hackethal's intramedullary nailing technique.

**Methods:**

In the time period from 2001 to 2010 156 patients with diaphyseal humeral fractures were treated surgically using Hackethal's technique. Six of them (3.8%) developed non-union. This group included three women and two men aged 63-69 years and one woman aged 37 years. The following parameters of the patients were recorded: age, gender, comorbidities, substance abuse, mechanism of injury, fracture type and location according to the AO/ASIF classification, and the operative technique.

**Results:**

A non-union developed in six patients treated with Hackethal´s method (3.8%). Five of six non-unions (83%) were observed in patients in their sixties. In the subgroup of sexagenarians, non-union developed in 20.8% of surgically treated patients, as compared to 3.8 % in entire group. In the union group, fractures have been caused by high energy trauma in 52% of patients. In patients who developed non-union, high energy trauma caused 67% of fractures. With correct surgical technique the development of a non-union was observed in 0.7% of patients, with incorrect technique in 35.7% (p<0.001).

**Conclusion:**

Treatment of diaphyseal humeral fractures with Hackethal's intramedullary elastic bundle nailing resulted in an overall high union rate. Factors contributing to the development of non-union were extension of this method to AO type B3 and C fractures and technical imperfection during implantation. Level of Evidence III, Prospective, Case-Control Study.

## INTRODUCTION

A variety of operative and non-operative treatment modalities exist for the treatment of diaphyseal humeral fractures. Hackethal,^1^ in 1961, described his elastic bundle nailing technique for the treatment of humeral diaphyseal fractures. Today, this technique is considered to be not sufficiently stable to achieve adequate bony union and has been largely replaced by solid intramedullary nailing and plate fixation.^2^ ^-^ ^7^ Nevertheless, with correct indication (simple AO A and B diaphyseal fractures) and adequate surgical technique (complete filling of the medullar cavity with the implants), this method meets the requirements of relative stability and biological osteosynthesis.^8^ ^-^ ^13^ Considering the good to excellent results achieved with this technique in simple fractures,^8^ ^,^ ^9^ ^,^ ^12^ we have decided to investigate factors that are influencing the development of humeral diaphyseal non-unions in patients treated with this method.

## MATERIALS AND METHODS

In the time period from January 2001 to December 2010, 156 patients were treated surgically using Hackethal´s elastic bundle nailing technique. Among these were 87 men (55%) and 69 women (45%). The mean patient age was 51.3 years old (range, 16 to 89 years). 

**Figure 1:**
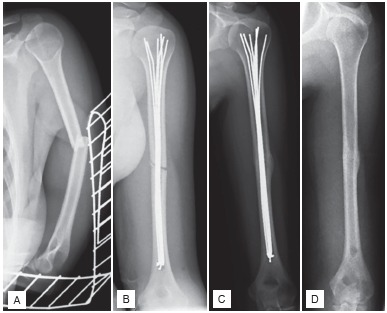
(A) Anteroposterior radiograph of a 31 year old female with an AO type 12-A3 mid-diaphyseal fracture of the humerus; (B) Postoperative radiograph showing complete filling of the medullary cavity with the elastic nails; (C) Follow-up at 10 months after surgery shows bony union; (D) Follow-up at one year after implant removal.

Surgery was indicated in patients with AO types A and B fractures of the humeral diaphysis. Patients with metaphyseal and epiphyseal fractures were excluded. (Figure 1) Indication was based on anteroposterior and lateral radiographs of the humerus. In a total of five cases, the indication was extended to multifragmentary (AO type C) diaphyseal fractures.

### Surgical technique

Surgery was performed according to the original description of Hackethal.^1^ Patients were placed in prone position with the ipsilateral arm placed on a radiolucent extension of the operating table. A 6 cm long dorsal midline approach was performed over distal humerus. After subcutaneous dissection, the triceps muscle was bluntly dissected and the distal humeral diaphysis was approached. The medullary cavity was opened dorsally and 5 - 6 mm proximally from the *fossa olecrani*. Trepanation was initiated by drilling three holes into the dorsal cortical bone which made up a triangle with side length of 1 cm and the base directed distally. The three drill holes were connected cautiously using a bone cutter. After concomitant reduction of fracture, 2 mm Kirschner wires were inserted with their blunt ends into medullar cavity. According to Hackethal's original description, the entire medullar cavity must be filled with implants; the mean number of wires was nine (ranging from 4 to 17). 

First, the Kirschner wires to be inserted were pre-bent before implantation, which facilitated their insertion and enabled positioning into different parts of humeral head. The final wires filling the medullar cavity were inserted without pre-bending. Wires were inserted until no further wire insertion was technically possible due to complete filling of bone cavity. Wire insertion was performed under fluoroscopic control in two perpendicular planes. The complete filling of the medullar cavity was also controlled fluoroscopically. Wire ends protruding after insertion distally were bent in angle of 90 degrees and shortened on level of dorsal bone cortex. (Figure 1) A 10Ch suction drainage was inserted in every case. None of patients did receive additional immobilization with a splint or orthosis. Surgery was performed by 14 different surgeons. Six of them were consultants and eight were residents under the direct supervision of a consultant.

### Postoperative care 

The suction drain was removed two days after surgery and passive range of motion (ROM) exercises were initiated as tolerated by the patients. Patients were discharged from hospital on the fourth day after surgery. After suture removal, 14 days after surgery, active ROM exercises were initiated with restriction of rotational movement of shoulder. Six weeks after surgery active ROM exercises were allowed without limitations. Clinical follow-up and radiographs were performed 6 weeks, 3, 6, 9 and 12 months after surgery. 

### Evaluation

The following parameters were observed with respect to their potential influence on non-union formation: patient age and sex, comorbidities, substance abuse, mechanism of injury, type and location of the fracture. Further, surgeon-related observed factors were indication and surgical technique, i.e. complete filling of medullar cavity with implants. Patients were grouped in those who developed non-union and those who did not. The differences between the groups were analysed with SPSS for Mac^(r)^ version 12.0 (SPSS Inc. Chicago, USA) using the t-test for patient age and Fisher's exact test or Chi square test for all other variables. A value of p<0.05 was considered statistically significant.

Due to the initiation year (2001), the study was not planned to be approved by the institutional ethics committee, therefore patients did not have to sign an informed consent.

## RESULTS

### Non-union

A non-union developed in six patients treated with Hackethal´s method (3.8%). This group comprised three women and two men aged 63-69 years and one woman aged 37. (Table 1) The latter had a higher biological age and poor compliance. The mean patient age in this group was 62 years. Revision surgery was indicated with the absence of bony union in control radiographs 4-9 months after the initial surgery and was performed in a time period from four months to three years after the primary surgery, according to patient compliance and preference. In four patients, bony union occurred after the first revision surgery, in one patient after the second revision surgery and one patient died from unrelated causes before bone healing could have occurred. (Table 2)

**Table 1:** Non-union patient group - basic data.

Patient Number	Gender	Age (years old)	Fracture location	Fracture type (AO)	Reason of non-union development
1	M	69	Middle diaphysis	12C3	Inadequate indication
2	F	69	Middle/proximal third of diaphysis	12B3	Incorrect technique
3	F	67	Middle diaphysis	12B2	Incorrect technique
4	M	66	Middle/proximal third of diaphysis	12A1	Incorrect technique
5	F	37	Proximal third of diaphysis	12C3	Inadequate indication, incorrect technique, noncompliance
6	F	63	Middle diaphysis	12B2	Incorrect technique

**Table 2:** Non-union patient group - treatment.

Patient Number	Time to revision surgery (months)	Method	Bony healing (months)	Comment
1	13	UHN*	13+6 M	
2	36	Hackethal	no	Died before healing occurred
3	4	UHN*	4+5 M	
4	15	Plate	15+5 M	Spastic quadruparesis
5	21	Locking plate	21+7 M	Noncompliance
6	5	UHN*	5+8 + 8 M	2^nd^ revision surgery using plate 13 months after injury

* UHN: Unreamed humeral nail.

Mean patient age in the union group was 50.8 years. The patients in the non-union group had a mean age of 62.0 years. With the numbers available, the difference between the groups was not significant (p=0.132). Five of six patients (83%) who developed non-union were in their sixties while in the union group only 19 of 150 patients (12.7%) were sexagenarians. The one patient who developed non-union at an age of 37 was judged to be much older biologically. Looking at the whole patient cohort, sexagenarians developed a non-union in five cases (20.8%).

Non-union developed more frequently in women. Among the patients with bony union, 43% were women, among those who developed non-union, 66% were women. The male to female ratio was reversed between the two groups. (Table 3) These differences between the groups, however, did not reach statistical significance.

**Table 3:** Comparison between the patient groups.

	Union group	Non-union group	*p* Value
Number of patients	150	6	-
Mean Age (range), years old	52.6 (16-89)	61.8 (37-69)	0.132
Male / female ratio	1.31	0.5	0.407
Smokers	37%	33%	1
Diabetics	10%	33%	0.129
High energy injury	52%	67%	0.684
Middle diaphyseal fracture	67%	67%	
Proximal diaphyseal fracture	26%	33%	
AO type A fracture	62%	17%	
AO type B fracture	36%	50%	
AO type C fracture	2%	33%	0.012
Incorrect technique	6%	83%	<0.0001

Sixteen patients were diabetics, two of them developed non-union; 58 patients were smokers, two of them developed non-union.

### Injury Mechanism

In the union group, the fracture had occurred after fall from standing height in 72 cases (48%). In the non-union group, fall from standing height had occurred only in two cases (33%). In the union group, a high energy trauma had occurred in 52%, in the non-union group the fracture had been caused more by high energy trauma in 67%. In particular, two women aged 37 and 67 years had a car accident, one 63 year old woman fell from a ladder and one 69 year old man fell from a roof. With the numbers available, the difference between the groups was not significant.

Thirty-four patients (22% of the entire group) were drunk at the time of injury, none of them developed a non-union.

The fracture location at the humeral diaphysis did not differ between the union and non-union groups. In both groups fractures were located in the mid-diaphysis of the humerus in 67%. Similarly, the fracture was located in the proximal third and at the junction between the proximal and middle third of the diaphysis in 39 patients from the union group (26%) and two patients from the non-union group (33.3%). 

A non-union developed more frequently in patients with more complex fractures (type B according to the AO classification). In the union group, 54 patients had suffered an AO type B fracture (36%). In the non-union group, three of six patients (50%) had suffered an AO type B fracture. Only one patient (17%) in the non-union group had an AO type A fracture, compared to 93 patients (62%) in the union group. Taken together, one of 94 patients (1.1%) with an AO type A fracture and three of 57 patients (5.3%) with an AO type B fracture developed non-unions.

### Indication and Technique

**Figure 2:**
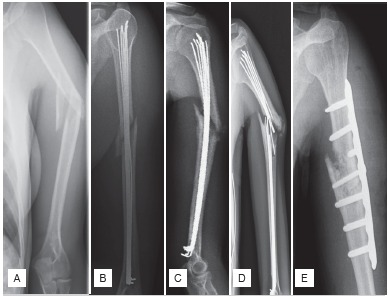
(A) Anteroposterior radiograph of a 37 year old female with an AO type 12-C3 mid-diaphyseal fracture of the humerus. (B) A radiograph obtained two days after surgery demonstrates that the medullar cavity is filled incompletely with implants and a slight distraction of the fragments; (C) Follow-up at 6 months after surgery shows no signs of bony consolidation with slight dislocation; (D) Follow-up radiograph at 18 months shows hypertrophic non-union with further dislocation and hardware failure. Non-union was treated surgically with removal of the elastic nails, resection of the pseudoarthrosis and interlocking plate fixation with cancellous bone grafting from the iliac crest; (E) At 21 months (3 months after revision surgery), union was finally obtained.

In the non-union group, two patients (33%) underwent surgery for a comminuted fracture (AO type C3). One of these patients was initially judged to have an AO B2 type fracture. The diagnosis was corrected perioperatively to AO type C3, but the surgical technique has not been changed. (Figure 2) In the union group, the indication was extended to AO type C fractures in three young patients (2%). These have healed without any complications. Correct indication of the technique with limitation to AO type A and B fractures has led to the development of a non-union in four out of 151 (2.6%) patients, while inadequate indication led to the development of a non-union in two out of five (40%) patients with AO type C fracture. The difference between the two groups was statistically significant (p=0.012). 

In five of six patients (83%) who developed non-union, the medullary cavity had been filled incompletely with implants. In one patient (17%) additionally a significant distraction of fragments persisted after surgery. (Figure 2) In the union group the medullary cavity had been filled incompletely with implants in nine of 150 cases (6%). Hence, with correct surgical technique, development of non-union was observed in only one out of 142 patients (0.7%), while technical errors led to the development of non-unions in five out of 14 patients (35.7%) (p<0.001).

## DISCUSSION

Hackethal's elastic bundle nailing technique is generally considered today to be not sufficiently stable to achieve adequate bony union in the treatment of humeral diaphyseal fractures and has been largely replaced by solid intramedullary nailing and plate fixation.^2^ ^-^ ^7^ In the pertinent literature, non-union rates between 1 and 9 % have been reported with Hackethal´s technique.^2^ ^,^ ^7^ ^,^ ^8^ ^,^ ^10^ ^,^ ^12^ ^,^ ^13^ Such a large variation is likely caused by different adherence of the authors to basic principles of indication and surgical technique as laid out by Hackethal^1^ in his original description. The main principle of this technique is to fill the entire medullary cavity with implants. The lowest incidence of non-unions (1%) was observed by Špáta et al.,^12^ who identified technical errors of the surgeon as a cause of the development of non-unions. Peter et al.,^10^ with the highest incidence of non-unions (9%), had filled the medullary cavity with only four or five 2-3 mm diameter Kirschner wires. Hackethal^1^ regards this amount as insufficient for the majority of humerus bones. The 3.8% incidence of non-unions in our 156 patient study compares favourably with the numbers from other studies. When analysing the cases with non-union, we could confirm a correlation between the development of non-union and inadequate surgical technique, i.e., incomplete filling of the medullary cavity with implants and additional persisting fragment diastasis after surgery. Technical errors can paradoxically be seen, despite the relative technical simplicity of this technique and are probably caused by its underestimation.^10^ ^,^ ^12^ ^,^ ^14^ When excluding the cases with inadequate technique, the overall non-union rate in our study would have dropped to 0.7%.

An insignificantly higher incidence of non-unions was seen in elderly patients with high-energy trauma. This is reflected by a higher proportion of more complex fractures (AO types B and C) caused by these injuries in the non-union group. Only one patient with an AO type A fracture (1.1%) developed non-union, compared to 5.3% of patients with AO type B and 40% of patients with AO type C fractures. In accordance with Hackethal's original principles,^1^ more complex fractures with extensive comminution (AO type C) should not be treated using this technique.^9^ Treating these fractures with more stable techniques (interlocking nails, locking plates) may be prone to more complications than elastic bundle nailing, but results in higher rates of union in more complex fractures.^3^ ^-^ ^7^ ^,^ ^11^ ^,^ ^13^ ^,^ ^15^ ^-^ ^23^

In the present study, non-union developed insignificantly more frequently in women and elderly patients aged between 60 and 70 years. Patients in this age group, particularly women, are more likely to have osteoporosis and other comorbidities resulting in poorer bone stock and therefore are more prone to complications. Treatment should be tailored individually in elderly and polymorbid patients and stability of fixation must be weighed against morbidity of the surgical approach. If the perioperative risk appears reasonable, more stable fixation methods should be considered. 

Several studies have identified diabetes and smoking as independent risk factors for the development of non-unions.^24^ ^,^ ^25^ Interestingly, none of the patients who were drunk at the time of injury and showed clinical signs of chronic alcohol abuse developed non-union. Again, the choice of the fixation method has to be guided individually by the surgeon's estimate of the perioperative risk of the approach to these fractures and patient compliance to avoid complications.

Our study has several limitations. One is the low number of non-unions in our study group. However, the whole cohort of 156 patients is in line with those reported in earlier studies and the total number of non-unions is limited by the relatively low incidence of this complication. Another limitation is the relatively short follow-up and the lack of clinical data. We believe this is acceptable, given the fact that the main outcome measure of this study was bony union and the main study question was which patient- and surgeon-related factors contributing to the development of non-union. As study strengths, there is the prospective analysis and the 100% follow-up of the entire patient cohort.

## CONCLUSIONS

Hackethal´s elastic bundle nailing technique is a relatively simple and safe method for the treatment of mid-diaphyseal humeral fractures. A high union rate can be achieved when adhering to the basic principles, as stated by its author. The first is the limitation of indication to diaphyseal fractures without a comminution zone (AO types A1-3 and B1-2), the second is a precise surgical technique with complete filling of medullary cavity with implants. In the present study, both inadequate indication and inadequate technique were associated with a significantly increased risk of non-union. 
